# Factors Impacting Meat Product Quality: From Farm to Table

**DOI:** 10.3390/foods15091533

**Published:** 2026-04-28

**Authors:** Leilson R. Bezerra, Cláudio V. D. M. Ribeiro

**Affiliations:** 1 Department of Animal Science, Federal University of Campina Grande, Patos 58708110, Brazil; 2 Department of Animal Science, Federal University of Bahia, Salvador 40170110, Brazil

## 1. Introduction

Meat quality is a complex and dynamic trait, resulting from the integration of biological, nutritional, environmental, and technological factors operating across the entire production chain, from primary production systems to consumer handling. Traditionally, meat quality has been defined by sensory attributes such as tenderness, juiciness, and flavor, as well as physicochemical parameters including pH, color, water-holding capacity, and lipid stability. However, contemporary perspectives have expanded this definition to encompass nutritional value, safety, shelf life, and even health-related properties, reflecting the evolving expectations of consumers and regulatory frameworks.

At the production level, animal genetics, feeding strategies, and management practices play a central role in determining muscle growth, fat deposition, and biochemical composition, which directly influence meat quality traits. Nutritional interventions, particularly those involving functional feed additives, lipid supplementation, and rumen-protected nutrients, have gained prominence as tools to modulate metabolic pathways, enhance fatty acid profiles, and improve oxidative stability. These strategies are increasingly aligned with the concept of precision nutrition, aiming to optimize animal performance while simultaneously improving product quality and sustainability.

Equally important are pre-slaughter conditions, including handling, transportation, and stress exposure, which can significantly affect muscle metabolism and post-mortem biochemical processes, leading to quality defects such as pale, soft, and exudative (PSE) or dark, firm, and dry (DFD) meat. These effects highlight the critical role of animal welfare as both an ethical and technological determinant of meat quality.

Advances in omics technologies, such as genomics, metabolomics, and transcriptomics, have provided unprecedented insights into the molecular mechanisms underlying meat quality, including flavor development, lipid metabolism, and oxidative processes. In parallel, the growing recognition of the gut microbiome as a key regulator of host metabolism has opened new avenues for understanding how microbial communities influence muscle composition and, ultimately, meat characteristics.

Post-harvest, technological innovations in processing, preservation, and packaging are essential to maintaining and enhancing meat quality. Emerging approaches, including superchilling, modified atmospheres, nanotechnology-based encapsulation, and functional coatings, are being developed to extend shelf life, control oxidation, and ensure microbial safety, while preserving sensory and nutritional attributes.

Importantly, meat quality must also be evaluated in the context of human health. Increasing attention has been given to the role of lipid oxidation products, protein oxidation, and bioactive compounds during digestion, linking meat consumption to metabolic and oxidative responses in the human body. This perspective reinforces the need for an integrated “farm-to-table” approach that considers not only production efficiency but also the nutritional and health implications of meat products.

In this context, this Special Issue aims to provide a comprehensive and multidisciplinary overview of the factors influencing meat quality, bringing together studies that explore innovations in animal nutrition, physiology, processing technologies, and food science. By integrating knowledge across these domains, the contributions in this issue advance our understanding of how to produce high-quality, safe, and sustainable meat products that meet the demands of modern consumers.

## 2. An Overview of the Published Articles

The ten articles included in this Special Issue collectively provide a comprehensive and interconnected perspective on the determinants of meat quality, highlighting how biological, nutritional, and technological factors converge along the production chain.

At the animal level, emerging evidence emphasizes the interaction between physiology, behavior, and metabolism as key drivers of meat quality. Zhang et al. [1] demonstrated that behavioral traits associated with physical activity are closely linked to improvements in meat characteristics in Qingyuan partridge chickens, an effect partially mediated by shifts in gut microbiota and metabolic pathways. This finding aligns with the broader concept that host–microbiome interactions play a pivotal role in modulating muscle development and composition.

Complementing this biological perspective, several studies explored the impact of nutritional strategies on meat quality. Jerónimo et al. [2] showed that high-forage, low-starch, high-oil diets can enhance the fatty acid profile of lamb meat without compromising growth performance, although with some trade-offs in feed efficiency. Similarly, Inô et al. [3] demonstrated that the use of rumen-protected lysine, particularly when combined with tannins, improves the concentration of health-promoting fatty acids such as conjugated linoleic acid and omega-3, while also enhancing oxidative stability. Together, these studies reinforce the role of precision nutrition as a central tool for improving both the nutritional value and technological quality of meat.

The biochemical and molecular basis of these qualitative attributes is further elucidated through advanced analytical approaches. Huang et al. [4] applied metabolomics and transcriptomics to reveal the metabolic pathways underlying flavor development in sheep meat, particularly those associated with amino acid and lipid metabolism. These findings provide mechanistic support for the nutritional and physiological effects observed in production systems, linking metabolic regulation to sensory outcomes.

In parallel, the importance of oxidative processes, both during processing and digestion, is highlighted by Ali et al. [5], who demonstrated that lipid and protein oxidation are strongly influenced by muscle type and production system. This study bridges the gap between meat processing and human health, emphasizing that meat quality must also be evaluated in terms of its post-consumption biochemical behavior.

From a technological standpoint, innovations in processing and preservation are essential to maintaining and enhancing meat quality. Ouyang et al. [6] demonstrated that modified starches can significantly improve texture, water retention, and oil absorption in processed meat products, particularly under freezing and reheating conditions. Similarly, Wang et al. [7] highlighted the effectiveness of superchilling technology in controlling microbial growth and oxidative degradation, thereby extending shelf life and preserving sensory attributes.

The integration of advanced technologies into meat production is further illustrated by Alvarenga et al. [8], who reviewed the potential of multimodal imaging techniques for the non-invasive prediction of key quality traits such as glycogen and intramuscular fat. These tools represent a critical step toward precision livestock production, enabling real-time decision-making and quality control.

In addition, the incorporation of bioactive compounds into meat systems has gained increasing attention. Oliveira et al. [9] discussed the use of essential and edible oils as natural preservatives, emphasizing the role of nanoencapsulation in enhancing their stability, bioavailability, and functional efficacy. In a similar context, Mendes et al. [10] reviewed the use of *Chlorella vulgaris* as a functional feed additive, demonstrating its potential to improve oxidative stability and nutritional quality, while also addressing practical challenges related to digestibility and optimal inclusion levels.

Collectively, these studies illustrate the transition of meat science toward a holistic and multidisciplinary framework, where animal biology, nutrition, processing technologies, and consumer health are intrinsically interconnected. This integrative approach is essential for advancing sustainable and high-quality meat production systems in response to evolving global demands.

## 3. Conclusions

The contributions presented in the Special Issue “Factors Impacting Meat Product Quality: From Farm to Table” reinforce the concept that meat quality is not determined by isolated factors but rather by the integration of multiple processes along the production chain. Advances in nutrition, particularly through functional ingredients and protected compounds, combined with emerging technologies such as omics approaches, imaging systems, and innovative preservation methods, are reshaping the field of meat science.

Importantly, these studies highlight the growing need to align production efficiency with sustainability and consumer health, emphasizing clean-label solutions, improved nutritional profiles, and reduced environmental impact. The integration of multidisciplinary approaches will be essential to address future challenges and to support the development of more resilient and sustainable meat production systems.

This Special Issue contributes to consolidating current knowledge while also identifying new research directions, particularly in the areas of precision nutrition, microbiome interactions, and advanced processing technologies. Ultimately, bridging the gap between fundamental science and industrial application will be key to delivering high-quality meat products that meet the expectations of modern consumers.
